# Protecting child and adolescent mental health in an uncertain future: commentary on Jaffee and colleagues' Annual Research Review – ‘Cash transfer programs and young people's mental health: a review of studies in the United States’

**DOI:** 10.1111/jcpp.14138

**Published:** 2025-02-21

**Authors:** Lucie Cluver

**Affiliations:** ^1^ Department of Social Policy and Intervention University of Oxford Oxford UK; ^2^ Department of Psychiatry and Mental Health University of Cape Town Cape Town South Africa

**Keywords:** cash transfer, Intervention, mental health, poverty, social policy

## Abstract

Jaffee and colleagues present a masterful review of the evidence for the impacts of cash transfer programmes on child and adolescent mental health in the United States. While global meta‐analyses find evidence of effectiveness, Jaffee and colleagues highlight the limited number of studies in Northern America, but find overall results indicating small but meaningful effect sizes on improving emotional and behavioural health, and greatest impacts for the poorest families.

Jaffee, Lin, Fowle, and Reina (2024) present a masterful review of the evidence for the impacts of cash transfer programmes on child and adolescent mental health in the United States. While global meta‐analyses find evidence of effectiveness, Jaffee and colleagues highlight the limited number of studies in Northern America, but find overall results indicating small but meaningful effect sizes on improving emotional and behavioural health, and greatest impacts for the poorest families.

Their findings from the United States share key similarities with those from low‐ and middle‐income countries: that cash transfers have the greatest impacts when they are continuous and consistent (rather than one‐off payments), and that they only improve mental health as long as they are still being delivered. These findings have important implications for identifying mechanisms of effect: just as children need stable caregiving and stable access to education, they also need stable economic security to support their emotional and behavioural health. If a child does not know whether next month their family will be homeless or hungry, then anxiety and depression are common, and treatment for those mental health conditions may benefit most from addressing their root causes in poverty.

The authors also highlight that child or adult psychopathology has not typically been a target of poverty reduction programmes. This conclusion is accurate, but perhaps also disguises the important strengths of such programmes. There is substantial evidence from high‐, low‐ and middle‐income settings that cash transfers reduce a range of negative outcomes for children and families, including child undernourishment, education loss and parental mental health distress—also highlighted in Jaffee's review—parent and child substance use, sexual risk behaviours and violence against children (e.g. Machado et al., 2024; Rawlings & Rubio, 2003). All of these negative outcomes are established predictors of lifetime psychopathology and suggest that there may be long‐term benefits of cash transfers on mental health, via these mediators, that remain largely untested in the United States.

This wider range of benefits for children and families may also be important for our conceptualisation of scaled‐up interventions. Low‐ and middle‐income settings are characterised by limited state provision of services, and overburdened health and community workforces. In these contexts, there is increasing focus on services that have simultaneous benefits across multiple domains—such as health, education and sexual risk reduction—and also on those services that have effects on multiple people within households or communities (Cluver et al., 2019). These ‘accelerator’ or ‘multiplier’ services require less complex service provision and potentially allow wider reach in constrained financial and human resource settings. It is also recognised that in contexts of limited government services, services that strengthen families to provide nurture and support become increasingly important in protecting children, as they provide the most proximal and consistent care (Desmond et al., 2022).

Cash transfers have the strongest evidence for such accelerator effects. Studies in lower income countries have shown similar impacts across conditional and unconditional cash transfers, with very much lower delivery costs for unconditional cash transfers (Baird, Garfein, Mcintosh, & Ozler, 2012). This strategy has led to their adoption as a programmatic priority by the World Bank, UNICEF, World Food Programme and governments across Africa, Latin America and Asia, and increasingly as anticipatory or responsive interventions in humanitarian disasters (Van Kim Robin et al., 2022). Despite this widespread acceptance of the value of cash transfers, access in low‐ and middle‐income countries still remains low, at less than one in 10 of children aged 0–15 estimated to receive a cash child benefit, based on UNICEF's Child Benefit Tracker.

The United States is likely to face a challenging next few years for child and adolescent mental health. There is now strong evidence of child mental health distress related to climate change and exposure to climate‐related disasters, which are increasing across the country: the past year has seen wildfires in Los Angeles and New Mexico, over 1700 tornados, winter storms across the Eastern US and Hurricanes Helene and Milton. There is also increasing evidence of child and adolescent psychological distress associated with increased mobile internet access, including online childhood sexual abuse and exploitation (Finkelhor, Turner, & Colburn, 2024). Demographic trends also show increasing risks for children's care stability, for example rising rates of parental death due to COVID‐associated orphanhood, parental substance use and suicide (Villaveces et al., 2025). All of these adverse developments suggest a need for population‐level prevention and care for child and adolescent mental health.

However, such population‐level care raises questions about money and human resources. An important question for the United States is whether there will be a move towards lower levels of government provision of services for children, reduced health and welfare workforces. If so, then child mental health will likely receive substantially less funding, resulting in less access to mental health professionals. While there will always be a need for mental health professionals and healthcare‐based services, the United States with rising child mental health needs and constrained funding may also benefit from looking towards models of provision from similar contexts in low‐ and middle‐income countries.

Accordingly, and unfortunately, these aforementioned trends suggest that the findings of Jaffee and colleagues' review will come into even more importance. They find beneficial impacts of unconditional, consistent, regular payments, particularly for the poorest families. They highlight the need for cash transfers to be of sufficient value that they are able to mitigate material hardship and suggest behavioural nudges in labelling cash transfer programmes as intended to benefit children. The authors also mention the potential value of combining cash transfers with mental health interventions, with upcoming trial results that will inform this recommendation. It is worth noting that quasi‐experimental evidence finds multiple benefits—including mental health benefits—for children and parents of cash support combined with positive parenting (Sherr et al., 2020), suggesting opportunities for considering a range of ‘cash plus’ combinations for vulnerable families. As cash transfers are increasingly able to be delivered digitally, and recent studies show the effectiveness of app‐based or digitally‐facilitated mental health and parenting interventions, there may also be new possibilities for lower‐cost combinations of cash plus care that can support child mental health at the scale that it is needed.

Jaffee and colleagues conclude with the need for more robust research in the US, including testing of hypothesised mediators of programme effects. Poverty alleviation may not have been a traditional focus of psychological or psychiatric researchers, nor of its journals. In commissioning this Annual Research Review, the *Journal of Child Psychology and Psychiatry* has demonstrated the need for us to think more broadly about interventions for child and adolescent mental health in the United States, to rapidly build a strong evidence base and to be strategic in planning for children in a complex and challenging next decade.
